# The Isoquinoline Alkaloid Dauricine Targets Multiple Molecular Pathways to Ameliorate Alzheimer-Like Pathological Changes *In Vitro*

**DOI:** 10.1155/2018/2025914

**Published:** 2018-07-02

**Authors:** Pan Liu, Xiao Chen, Haizhe Zhou, Liqun Wang, Zaijun Zhang, Xiaohu Ren, Feiqi Zhu, Yi Guo, Xinfeng Huang, Jianjun Liu, Peter S. Spencer, Xifei Yang

**Affiliations:** ^1^College of Pharmaceutical Engineering & Life Science, Changzhou University, Changzhou 213164, China; ^2^Key Laboratory of Modern Toxicology of Shenzhen, Shenzhen Center for Disease Control and Prevention, Shenzhen 518055, China; ^3^Department of Encephalopathy, Shaanxi University of Chinese Medicine, Xianyang, Shanxi 712000, China; ^4^Institute of New Drug Research and Guangzhou Key Laboratory of Innovative Chemical Drug Research in Cardio-cerebrovascular Diseases, Jinan University College of Pharmacy, Guangzhou 510632, China; ^5^Cognitive Impairment Ward of Neurology Department, The 3rd Affiliated Hospital of Shenzhen University, Shenzhen 518055, China; ^6^Department of Neurology, Second Clinical College, Jinan University, Shenzhen 518020, China; ^7^Department of Neurology, School of Medicine, Oregon Institute of Occupational Health Sciences, Oregon Health & Science University, Portland, OR 97239, USA

## Abstract

Alzheimer's disease (AD), the most common neurodegenerative disease, has no effective treatment. Dauricine (DAU), a benzyl tetrahydroisoquinoline alkaloid isolated from the root of *Menispermum dauricum* DC, reportedly has neuroprotective effects in cerebral ischemia. Here, we investigated the effects of DAU on N2a cells stably transfected with Swedish mutant amyloid precursor protein (N2a/APP), an AD-like cell model. ELISA and Western blot analysis revealed that DAU inhibited APP processing and reduced A*β* accumulation. In addition, DAU ameliorated tau hyperphosphorylation via PP2A, p35/25, and CDK5 pathways in N2a/APP cells. The amelioration of tau hyperphosphorylation by DAU was also validated in HEK293/Tau cells, another cell line with tau hyperphosphorylation. Proteomic analysis revealed 85 differentially expressed proteins in the lysates between the wild-type N2a cells (N2a/WT) and the N2a/APP cells in the presence or absence of DAU; these were classified into 6 main categories according to their functions: endoplasmic reticulum (ER) stress-associated proteins, oxidative stress-associated proteins, cytoskeleton proteins, molecular chaperones, mitochondrial respiration and metabolism-related proteins, and signaling proteins. Taken together, we demonstrated that DAU treatment reduces AD-like pathology, thereby suggesting that DAU has potential therapeutic utility in AD.

## 1. Introduction

Alzheimer's disease (AD), a progressive and irreversible neurodegenerative disorder, contributes to individual morbidity and mortality and burdens the social healthcare system [1, 2]. AD has complex neuropathological features, but neurofibrillary tangles consisting of abnormal phosphorylated tau and neuritic amyloid *β* (A*β*) plaques are hallmarks of the disease. The approved medications for AD show consistent but modest clinical effects [3, 4]; on the contrary, hundreds of trials with candidate AD drugs have been terminated because they were clinically ineffective. A medication that can prevent, delay, or reverse the disease has yet to be discovered.

**Figure 1:**
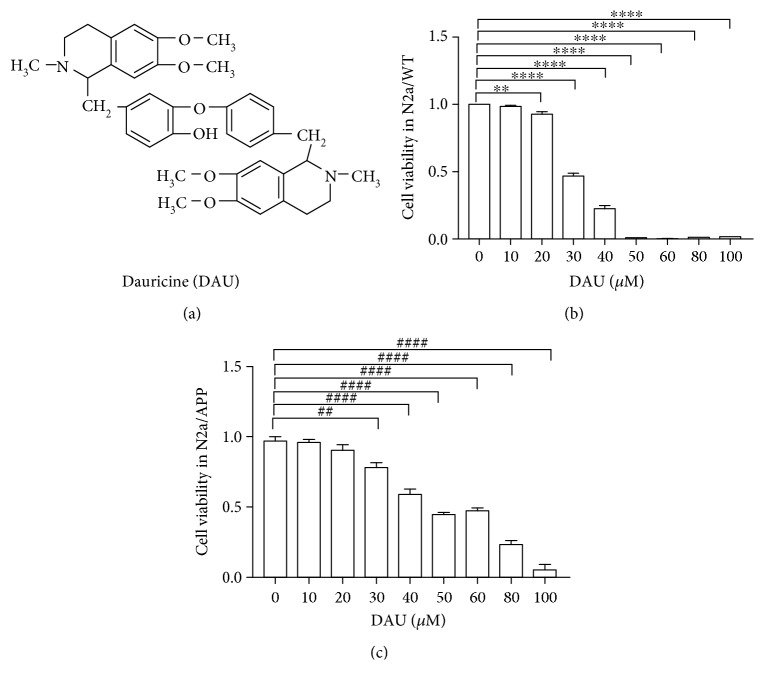
DAU has low cytotoxicity to N2a/WT and N2a/APP cells. (a) Chemical structure of DAU. (b) Cell viability of N2a/WT with respect to DAU treatment. (c) Cell viability of N2a/APP with DAU treatment. *N* = 3. ^∗∗^ *P* < 0.01 and ^∗∗∗∗^ *P* < 0.0001 compared with N2a/WT cells treated with vehicle. ^##^ *P* < 0.01, ^####^ *P* < 0.0001 compared with vehicle-treated N2a/APP cells.

Bisbenzylisoquinolines form a class of natural products with a therapeutic potential for neurodegeneration [5]. Dauricine (DAU) is a bisbenzylisoquinoline alkaloid derivative (Figure 1(a)) extracted from the rootstock of *Menispermum dauricum* DC, a traditional medicine listed in the Chinese Pharmacopoeia. The neuroprotective effects of DAU have been widely reported. DAU inhibited apoptosis of a transient focal cerebral ischemia model in part via a mitochondrial pathway [6]. DAU protected cortical neurons from ischemia by inhibiting entry of extracellular Ca^2+^ and intracellular release of Ca^2+^ from endoplasmic reticulum [6]. DAU reduced neurological deficits, diminished DNA fragmentation, increased Bcl-2 expression, and reduced Bax expression in ischemic cerebral infarcts via modulation of Bcl-2 family proteins [6]. DAU attenuated tau hyperphosphorylation by promoting the release of bradykinin, which raised intracellular neuronal calcium [7]. Another bisbenzylisoquinoline alkaloid, tetrandrine, has been reported to attenuate spatial memory impairment and hippocampal inflammation by inhibiting NF-*κ*B activation in a rat model of AD induced by A*β*_1–42_ [5]. However, the therapeutic potential of DAU has yet to be evaluated in a transgenic model of AD.

Given that bisbenzylisoquinolines are potential AD drug candidates, we examined the neuroprotective effects of DAU in a murine neuroblastoma cell line (N2a) stably transfected with the human Swedish mutant form of amyloid protein precursor (APP) [8]. By employing this well-studied cell model [9], which overexpresses APP and hyperphosphorylates tau, we found that DAU not only attenuated the level of tau hyperphosphorylation but also reduced A*β* plaque formation. Accompanying these changes, DAU altered the unfolded protein response, mitochondrial function, and clearance of reactive oxygen species.

## 2. Methods and Material

### 2.1. Reagents

DAU (stated purity ≥ 98%) was purchased from Shanghai Aladdin Biochemical Technology Co. Ltd. (CAS: 524-17-4, D115683, Shanghai, China). The purity of the DAU was confirmed by HPLC. The stock solution of DAU (10 mM) was prepared in DMSO (Thermo Fisher Scientific, Waltham, MA, USA) and was used directly. The antibodies used in this study are listed in Table 1.

### 2.2. Cells and Cell Culture

Wild-type murine neuroblastoma Neuro2a cells (N2a/WT) were purchased from the Cell Bank of China (CODE: IFO50495, Shanghai, China). N2a cells stably transfected with human APP Swedish mutant (N2a/APP) and Human Embryonic Kidney 293 cells stably transfected with tau protein (HEK293/Tau) were gifts from Professor Jian-zhi Wang (Tongji Medical School, Wuhan, China) [8–10]. The cells were cultured in the following medium with 5% CO_2_ and at 37°C: Minimum Essential Medium Eagle (MEM) (Grand Island, NY, USA) with 10% fetal bovine serum (FBS, Grand Island, NY, USA) for N2a/WT cells; Dulbecco's modified Eagle's medium (DMEM, Grand Island, NY, USA) with 10% FBS for HEK293/Tau cells; and a medium containing 42% DMEM and 50% Opti-MEM, complemented by 8% FBS for N2a/APP cells. Geneticin (0.2 g/L) (Grand Island, NY, USA) was dissolved in a medium to select transfected N2a/APP and HEK293/Tau cells.

### 2.3. Cell Viability Assay

After subculture, the N2a/APP cell suspension was placed on a 96-well tissue culture plate. Each well of the plate contained 10^4^ cells in 100 *μ*L cell culture medium. After 16 h in 5% CO_2_ at 37°C, the medium was removed and replaced with 200 *μ*L cell culture medium with DAU or vehicle (0.5% DMSO) (Thermo Fisher Scientific, Waltham, MA, USA). After another 24 h in 5% CO_2_ at 37°C, the medium was decanted and replaced by 100 *μ*L cell culture medium with 10 *μ*L cell counting kit-8 solution (Dojindo Laboratories, Kumamoto, Japan) and incubated for 1 h. The plate was read by a plate reader (TECAN Group Ltd., San Jose, CA, USA) at 450 nm. Cell viability was calculated as the absorbance of the well with cell and cell culture medium minus the well with cell culture medium only. The relative cell viability was the viability of the treated cell normalized by the viability of the control (vehicle).

### 2.4. ELISA of A*β*_1–40_ and A*β*_1–42_

The ELISA kits for A*β*_1–40_ and A*β*_1–42_ (R&D Systems, Minneapolis, MN, USA) were used according to the manufacturer's protocol. N2a/WT and N2a/APP cells were treated with DAU or vehicle in 25 cm^2^ culture flasks for 24 h (Grand Island, NY, USA). Cells and culture medium were collected separately. The cells were lysed in 200 *μ*L IP buffer (Beyotime, Beijing, China) with protease and phosphatase inhibitor cocktail (Thermo Fisher Scientific, Rockford, IL, USA) on ice for 20 min and centrifuged at 18,000 at 4°C for 20 min. The resultant supernatant was diluted 40 times before assay, and the cell culture medium was used directly. A standard curve of A*β*_1–40_ or A*β*_1–42_ was built, and the A*β*_1–40_ or A*β*_1–42_ in the samples was calculated and normalized by total protein content determined with a Pierce™ BCA protein assay kit (Thermo Fisher Scientific, Rockford, IL, USA).

### 2.5. Western Blot Analysis

After 24 h treatment with DAU or vehicle, cells were collected and lysed in 200 *μ*L of IP buffer with protease and phosphatase inhibitor cocktail on ice for 20 min and centrifuged at 14,000*g* at 4°C for 20 min. Supernatants were used for protein content determination and SDS-PAGE separation. The total protein content of each sample was determined with the Pierce BCA protein assay kit. Before loading onto the SDS-PAGE gel, samples were mixed with Pierce Lane Marker Reducing Sample Buffer (Thermo Fisher Scientific, Rockford, IL, USA) and denatured (boiled for 10 min). SDS-PAGE (10–12%) gels were used to separate target proteins and then transferred to polyvinylidene fluoride (PVDF) membranes (Merck Millipore Ltd., Merck KGaA, Darmstadt, GER). Membranes were blocked with nonfat milk powder dissolved in TBS-Tween 20 buffer for 2 h and then incubated with primary antibody (dilutions of the antibodies are listed in Table 1) at 4°C overnight. The membranes were washed and incubated with anti-mouse, anti-rabbit, or anti-goat IgG conjugated to horseradish peroxidase (HRPs) (1 : 3000) at room temperature (RT) for 1 h before development. Enhanced chemiluminescent solution (Thermo Fisher Scientific, Rockford, IL, USA) was applied for development. The densitometry of the blots was quantified by ImageQuant 1D software (GE. Healthcare, Pittsburgh, PA, USA).

### 2.6. Comparative Proteomics

#### 2.6.1. Protein Preparation and Labeling

After 24 h treatment with DAU or vehicle, cells were collected and lysed in 500 *μ*L DIGE-specific lysis buffer (7 M urea, 2 M thiourea, 30 mM Tris–HCl, 4% CHAPS, pH 8.5) on ice for 30 min. Ultrasonication (Fisher 550 Sonic Dismembrator, Pittsburgh, PA, USA) was applied to assist cell lysation. Samples were centrifuged at 20,000 *g* for 60 min. For each sample, 200 *μ*L of lysis buffer was added to the supernatant, and then the mixture was ultrafiltered in a centrifugal filter (Merck Millipore Ltd., Billerica, MA, USA) to remove salts. The protein solution was collected, and protein concentrations quantified were with a 2-D Quant kit (GE Healthcare, Chicago, IL, USA) according to the manufacturer's guidelines.

Fifteen protein samples were used for the proteomics study and for each treatment group, and three biological repeats were performed for each group. Each protein sample was diluted with lysis buffer to a final concentration of 5 *μ*g/*μ*L. The protein solution (5 *μ*L) was labeled with Cy3 (GE Healthcare, 25-8008-61) or Cy5 dye (GE Healthcare, 25-8008-62) in the dark for 30 min. Cy2 stained the internal standard that was pooled from 15 protein study samples. The reaction of protein labeling was quenched by adding 1 *μ*L of 10 mM lysine (Sigma-Aldrich, L5626), and three samples labeled with Cy2, Cy3, and Cy5 were mixed as a group. The mixture was then resolved in rehydration buffer (7 M urea, 2 M thiourea, 2% CHAPS, 2.8% DTT, 0.5% IPG buffer (pH 3–11 NL) and 0.002% bromophenol blue) to a final volume of 450 *μ*L prior to transfer onto immobilized pH gradient strips.

#### 2.6.2. 2-Dimensional Electrophoresis

The strips were first rehydrated and then isoelectric-focused (IEF) in Ettan IPGphor Isoelectric Focusing (IEF) System (GE Healthcare). After IEF, strips were allowed to stand at RT for 10 min. The focused strips were immediately equilibrated in a 15 mL reducing equilibration buffer of 6 M urea, 30% (*v*/*v*) glycerol, 2% (*w*/*v*) SDS, 75 mM Tris-HCl buffer (pH 8.8), and 1% (*w*/*v*) DTT (Sigma-Aldrich, St. Louis, MO, USA) for 15 min at RT on a shaking table and subsequently reequilibrated in the same buffer containing 6 M urea, 75 mM Tris–HCl buffer (pH 8.8), 30% (*v*/*v*) glycerol, 2% (*w*/*v*) SDS, and 4.5% (*w*/*v*) IAA (Sigma-Aldrich) afterwards. The equilibrated strips were loaded on the top of 12.5% SDS-PAGE gels and covered with 0.5% (*w*/*v*) ultralow melting point agarose sealing solution (25 mM Tris, 192 mM glycine, 0.1% SDS, 0.5% (*w*/*v*) agarose, 0.02% bromophenol blue). Protein separation in the second dimension employed an Ettan DALTsix Electrophoresis System (GE Healthcare) with the running buffer (25 mM Tris, 192 mM lycine, 0.1% SDS, pH 8.3) at 12°C by the following steps: 1 W/gel for 1 h, subsequently 11 W/gel for 5 h in the dark. Afterward, gels were immediately scanned in a Typhoon TRIO Variable Mode Imager (GE Healthcare) after a prescan at 1000 micrometer resolution to determine the optimum PMT voltage for each channel. Image acquisition was done with a resolution of 100 micrometers. To achieve variation in the signal across gels, the PMT was set to ensure the maximum pixel intensity of all gel images remained within a range of 40,000–60,000 pixels.

#### 2.6.3. Image Analysis

Following the manufacturer's instruction, DIGE gels were analyzed with the DeCyder software package (version 6.5 GE Healthcare, Milwaukee, USA). Each gel image was imported into the software and then individually processed with the differential in-gel analysis (DIA) and the biological analysis (BVA) modules to analyze protein spots. The volume of each protein spot in the Cy3 or Cy5 channels was normalized against the volume of the same Cy2 spot. The normalized volume of each spot was compared across the gels among the replicate groups. Differentially expressed protein spots (*P* < 0.05) were shortlisted for identification.

#### 2.6.4. In-Gel Tryptic Digestion

Replicate preparative gels of 1000 *μ*g of N2a/WT and N2a/APP cell proteins were prepared as for DIGE but without protein labeling. The gel was immersed overnight in dye (Coomassie blue solution containing 0.12% Coomassie brilliant blue G-250, 20% ethanol, 10% phosphoric acid, and 10% ammonium sulfate). Differential protein spots of interest identified by Decyder software analysis were manually excised from the stained gel. Gel pieces were destained and digested overnight at 37°C with 0.01 *μ*g/*μ*L trypsin (Promega Corp., WI, USA) as described by Robinson et al. [11]. The tryptic peptides were used for analysis by matrix-assisted laser desorption/ionization time-of-flight tandem mass spectrometry (MALDI-TOF-MS/MS) (SCIEX TOF/TOF™ 5800 System, AB SCIEX, Framingham, MA, USA).

#### 2.6.5. Mass Spectrometry and Database Searching

MALDI mass measurements were carried out with a Bruker Ultraflex III MALDI-TOF/TOF mass spectrometer (Bruker, Billerica, MASS, USA). For each protein sample, a total of 0.8 *μ*L peptide extract was used for MALDI-TOF-MS/MS analysis, and the peptide extract was cocrystallized with 0.8 *μ*L and 10 mg/mL *α*-cyano-4-hydroxycinnamic acid (CHCA) in 0.1% TFA, 50% acetonitrile (ACN) directly on the target, and dried at RT. The spectra were externally calibrated. MASCOT (Matrix Science, UK) was used for database searching against the SwissProt databases for murine cells proteins. The search was performed in the *Mus musculus* database and conducted with a tolerance on a mass measurement of 100 ppm in the MS mode and 0.5 Da in the MS/MS mode. Up to two missed cleavages per peptide were allowed. A fixed carbamidomethyl modification was taken into account. Protein MW and PI information were also considered to evaluate the protein identification based on the location of the excised protein spot from the 2-D gel.

#### 2.6.6. Immunocytochemistry

Cells were planted on a coverslip within a well of a 6-well plate. After the cells attached to the slip, they were treated with DAU or vehicle for 24 h. Cells on the coverslip were fixed in 4% polyaldehyde for 10 min at RT, permeabilized in 0.3% Triton X-100 in PBS for 30 min, and blocked with 5% BSA in PBST. After blocking, the coverslip was incubated with anti-8-OHdG (1 : 400) at 4°C overnight. After washing, the coverslip was incubated with anti-goat IgG conjugated to HRPs (1 : 200) (Santa Cruz Biotechnology, Santa Cruz, CA, USA) in the dark for 1 h. The cells on the coverslip were then stained with DAPI (4′,6-diamidino-2-phenylindole) for 5 min and developed with Fluo-Antifading Medium (Beyotime, Beijing, China). Cells were examined by laser confocal microscopy.

#### 2.6.7. Bioinformatics Analysis and Statistics

Functional annotation of differentially expressed proteins was performed with the Database for Annotation, Visualization and Integrated Discovery Resource (DAVID, https://david.ncifcrf.gov). Gene ontology (GO) terms for biological processes (BP), molecular functions (MF), and charts and cellular components (CC) were obtained with default statistical parameters.

Results were expressed as the mean ± SEM. One-way ANOVA was used to determine the statistical significance of differences among groups and following post hoc assessment by the Student-Newman-Keuls Method (GraphPad Prism 7.0, http://www.graphpad.com/). A *P* value less than 0.05 was considered statistically significant.

## 3. Results

### 3.1. DAU Has Low Cytotoxicity to N2a/WT and N2a/APP Cells

DAU is a bisbenzylisoquinoline alkaloid derivate (Figure 1(a)) extracted from the rootstock of *Menispermum dauricum* DC, a traditional medicine listed in the Chinese Pharmacopoeia. We investigated the cytotoxicity of DAU on both N2a/WT and N2a/APP cells using a 24 h cell-based assay. CCK-8, a water-soluble tetrazolium salt that is converted to a water-soluble formazan dye by living cell mitochondria, was exploited to examine cell viability. No significant inhibition of cell viability was observed in N2a/WT cells treated with less than 20 *μ*M DAU compared with vehicle-treated cells (Figure 1(b)). We did not observe obvious reductions of cell viability when the N2a/APP cells were treated with 10 *μ*M or 20 *μ*M DAU (Figure 1(c)). We therefore concluded that no significant cytotoxicity was induced by 24 h treatment of DAU even at the concentration of 20 *μ*M, the maximum concentration of DAU used in the following study.

### 3.2. DAU Inhibited APP Processing and A*β* Accumulation in N2a/APP Cells

**Figure 2:**
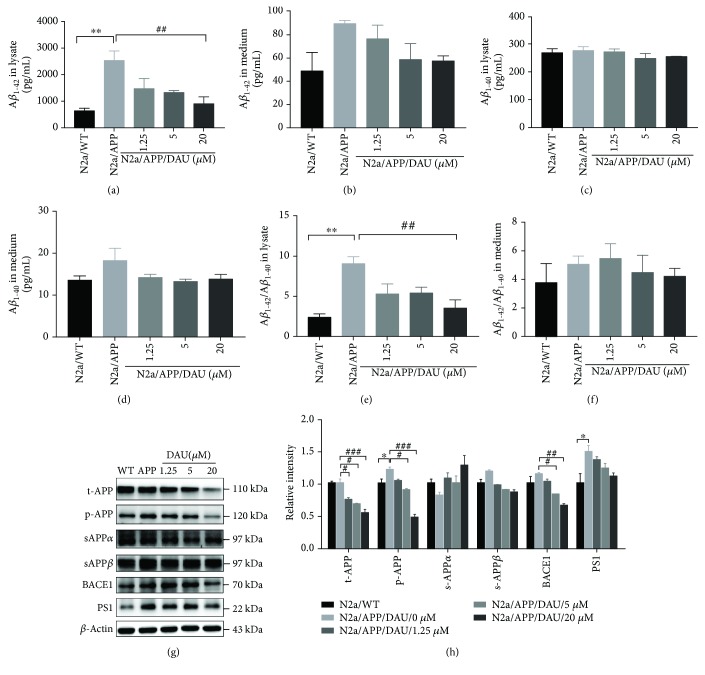
DAU inhibited APP processing and A*β* accumulation. Levels of A*β*_1–42_ (a, b), A*β*_1–40_ (c, d), and A*β*_1–42_/A*β*_1–40_ (e, f) of cell lysates (a, c, e) and cell culture media (b, d, f) as a function of DAU concentration were determined by ELISA. Levels of t-APP, p-APP, s-APP*α*, s-APP*β*, BACE1, and PS1 were determined by Western blot analysis (g, h). *β*-Actin was used as a loading control. *N* = 3. Data show the mean ± SEM. ^∗^ *P* < 0.05 and ^∗∗^ *P* < 0.01 compared to N2a/WT cells. ^#^ *P* < 0.05, ^##^ *P* < 0.01, and ^###^ *P* < 0.001 compared to untreated N2a/APP cells.

We then investigated the effect of DAU on A*β* generation with ELISA. The level of A*β*_1–42_ toxic fragments was significantly higher in N2a/APP cell lysates (2538 pg/mL versus 646.5 pg/mL, *P* = 0.0029) compared to N2a/WT cell lysates, and the A*β*_1–42_ level in N2a/APP cells treated with 20 *μ*M DAU was nearly three times lower (909.6 pg/mL versus 2538 pg/mL, *P* = 0.0085) (Figure 2(a)). The mean level of A*β*_1–42_ in N2a/APP cell culture medium was also higher than that of N2a/WT cells (89.21 pg/mL versus 48.71 pg/mL, *P* = 0.0996) (Figure 2(b)). On the contrary, there was no significant difference in the level of nontoxic amyloid A*β*_1–40_ in either the cell lysate (278.5 pg/mL versus 270.8 pg/mL, *P* = 0.9894) (Figure 2(c)) or cell culture medium (18.26 pg/mL versus 13.58 pg/mL, *P* = 0.1975) in N2a/WT or N2a/APP cells (Figure 2(d)). The ratio of A*β*_1–42_/A*β*_1–40_ in the lysates of N2a/APP cells was 3 times higher than that of N2a/WT cells (9.05 versus 2.41, *P* = 0.0026), and a comparable reduction was observed in the ratio of A*β*_1–42_/A*β*_1–40_ in N2a/APP cells treated with 20 *μ*M DAU lysates compared with the lysates from cells treated with vehicle (3.54 versus 9.05, *P* = 0.0099) (Figure 2(e)). A diagram of the ratio of A*β*_1–42_/A*β*_1–40_ (Figure 2(f)) shows a similar trend, but there was no difference between the groups.

The effect of DAU on APP processing was investigated further by Western blot analysis. N2a/APP cells had a significantly higher level of phosphorylated (amyloid precursor protein) APP and presenilin 1 (PS1) than N2a/WT cells (Figures 2(g) and 2(h)). The mean levels of *β*-secretase (BACE1) and insoluble *β*-secretase-cleaved amyloid precursor protein (sAPP*β*) were also higher in N2a/APP cells compared to N2a/WT cells. DAU-treated N2a/APP cells significantly decreased the expression of total APP, phosphorylated APP, and BACE1. DAU-treated N2a/APP cells also showed reduced mean levels of sAPP*β* and PS1, while the mean level of *α*-secretase-cleaved amyloid precursor protein (sAPP*α*) was higher. DAU-induced changes in APP processing appeared to correlate with changes in A*β* levels assayed by ELISA.

### 3.3. DAU Attenuated Tau Pathology via PP2A and p35/25 in Both N2a/APP Cells and HEK293/Tau Cells

**Figure 3:**
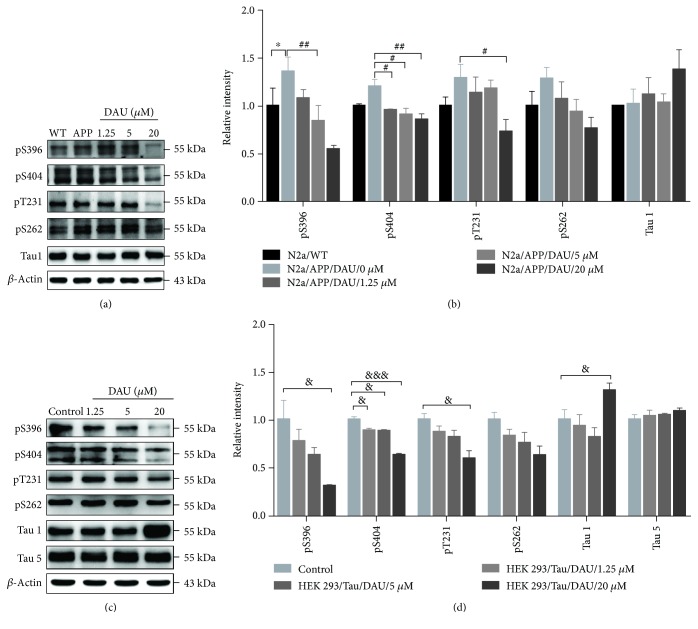
DAU attenuated tau phosphorylation in both N2a/APP cells and HEK293/Tau cells. Levels of phosphorylated tau and total tau in N2a/WT and N2a/APP cells (a, b) and in HEK293/Tau cells (c, d) as determined by Western blot analysis. *β*-Actin was used as a loading control. *N* = 3. Data show the mean ± SEM. ^∗^ *P* < 0.05 compared to N2a/WT cells, ^#^ *P* < 0.05 and ^##^ *P* < 0.01 compared to untreated N2a/APP cells, and ^&^ *P* < 0.05 and ^&&&^ *P* < 0.001 compared to untreated HEK293/Tau cells.

We next used Western blot analysis to investigate the effect of DAU on the tau pathology of N2a/APP cells. Vehicle-treated N2a/APP cells had significantly increased tau phosphorylation at serine 396 than N2a/WT cells (Figures 3(a) and 3(b)). The mean levels of phosphorylated tau at serine 404, serine 262, and threonine 231 sites were higher in N2a/APP cells compared to N2a/WT cells, while the mean level of dephosphorylated tau (Tau-1) was lower in N2a/APP cells compared to tau phosphorylation at serine 396, serine 404, and threonine 231 sites. The levels of phosphorylated tau at serine 404, serine 396, and threonine 231 were significantly reduced in DAU-treated N2a/APP cells compared with the vehicle-treated N2a/APP cells. The mean levels of phosphorylated tau at serine 262 were reduced in DAU-treated N2a/APP cells compared with the vehicle-treated N2a/APP cells, while mean levels of Tau-1 were increased in DAU-treated N2a/APP cells versus vehicle-treated N2a/APP cells. To verify the results from N2a/WT and N2a/APP cells, we examined the effects of DAU treatment on the phosphorylation of tau on HEK293/Tau cells that overexpresses tau. As shown in Figures 3(c) and 3(d), we observed similar reductions of tau phosphorylation (serine 396, serine 404, and threonine 231) and similar enhancement of the level of Tau-1. DAU did not alter the expression of total tau protein.

**Figure 4:**
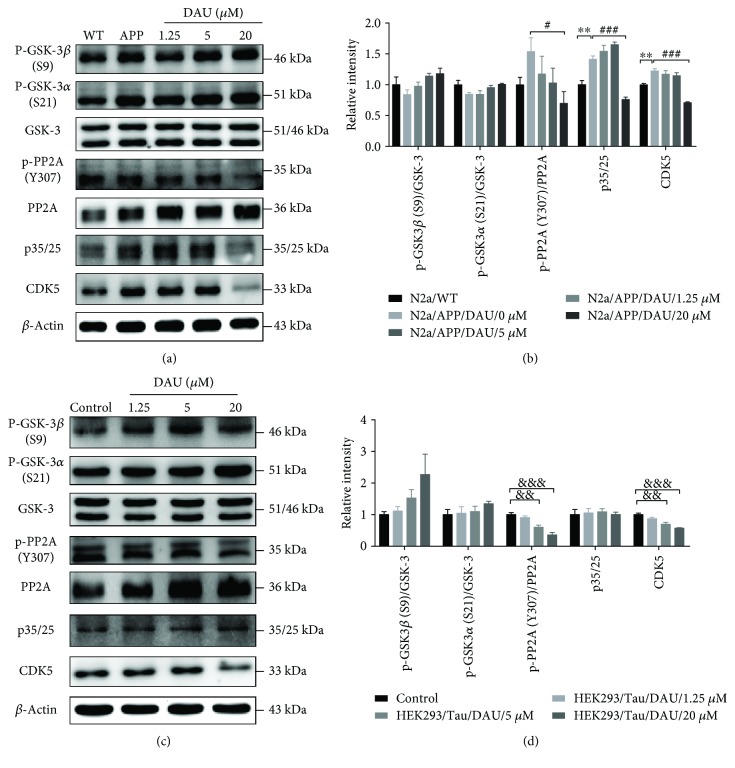
DAU ameliorated tau pathology via PP2A and p35/25 in both N2a/APP cells and HEK293/Tau cells. Phosphorylation of GSK3 and PP2A and levels of p35/25 and CDK5 in N2a/APP cells (a, b) and HEK293/Tau cells (c, d) as determined by Western blot analysis. *β*-Actin was used as a loading control. *N* = 3. Data show the mean ± SEM. ^∗∗^ *P* < 0.01 compared to N2a/WT cells, ^#^ *P* < 0.05 and ^###^ *P* < 0.001 compared to untreated N2a/APP cells, and ^&&^ *P* < 0.01 and ^&&&^ *P* < 0.001 compared to untreated HEK293/Tau cells.

While phosphorylation of tau can arise from the action of many catalyzing kinases and phosphatases [12], we investigated DAU-induced changes in those pathways shown to be key to tau phosphorylation, namely, glycogen synthase kinase-3*β* (GSK-3*β*), protein phosphatase2A (PP2A), and the p35/25 pathways. DAU treatment lowered the mean phosphorylation levels of glycogen synthase kinase-3*α* (GSK-3*α*) and GSK-3*β* in N2a/APP cells more than in N2a/WT cells (Figures 4(a) & 4(b)). The mean phosphorylation state of PP2A and the levels of p35/25 and cyclin-dependent kinase 5 (CDK5) were enhanced in N2a/APP cells compared with N2a/WT cells. DAU treatment enhanced the mean levels of both GSK-3*α* and *β* in N2a/APP cells compared with vehicle-treated N2a/APP cells. More importantly, 20 *μ*M DAU significantly decreased the phosphorylation of PP2A and levels of p35/25 and CDK5 in N2a/APP cells compared with vehicle-treated N2a/APP cells. As a supplemental validation, the phosphorylation of PP2A and levels of p35/25 and CDK5 in HEK293/Tau cells were similarly modulated by DAU treatment. Thus, DAU may ameliorate tau pathology via PP2A, p35/25, and CDK5, rather than GSK-3*β*.

### 3.4. DAU Modified Proteins That Involve Oxidative Stress, Mitochondrial Function, and ER Stress of N2a/APP Cells

**Figure 5:**
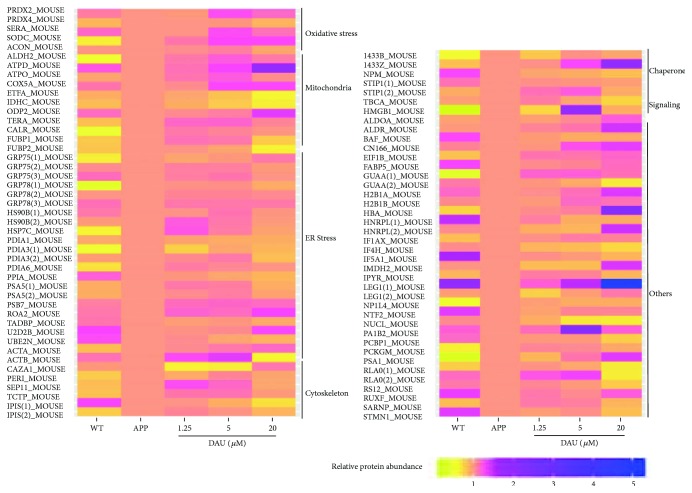
DAU caused differential expression of proteins in N2a/APP cells. DAU caused differential expression of 49 proteins including oxidative stress-associated proteins, endoplasmic reticulum (ER) stress-associated proteins, metabolism-associated proteins, cytoskeleton-associated proteins, molecular chaperones-associated proteins, signaling-associated proteins, and others. Protein expression was significantly altered in any four comparison pairs (N2a/APP versus N2a/WT, 1.25 *μ*M DAU versus N2a/APP, 5 *μ*M DAU versus N2a/APP, or 20 *μ*M DAU versus N2a/APP).

To explore molecular species affected by DAU treatment, we performed a comparative proteomic analysis using 2D-DIGE separation and MS identification. A total of 85 proteins in 2D-DIGE gels was significantly different in any four comparison pairs (N2a/APP versus N2a/WT, 1.25 *μ*M DAU versus N2a/APP, 5 *μ*M DAU versus N2a/APP, or 20 *μ*M DAU versus N2a/APP) (shown in Figure 5). Six protein categories were impacted by DAU treatment: endoplasmic reticulum (ER) stress-associated proteins, oxidative stress-associated proteins, cytoskeleton-associated proteins, molecular chaperones, mitochondrial respiration, and metabolism signaling proteins. In addition, we found some common proteins that were differentially expressed among the four comparison groups (Figure S1A and Tables S1–4). These differential proteins included signaling protein high-mobility group protein B1 (HMGB1) a mediator of neurite degeneration through the identification of the pathological signaling pathway in AD [13]; molecular chaperones (heat shock cognate 71 kDa protein (HSP7C)), a molecular chaperone and a member of the heat shock protein family that plays an integral role in the stress response [14]; translationally controlled tumor protein (TCTP), which has critical roles in the defense against oxidative and thermal stresses [15] (shown in Table S1); endoplasmic reticulum (ER) stress-associated protein (protein disulfide-isomerase A6 (PDIA6)), a protein related to ER stress that plays a critical role in most biological processes [16]; oxidative stress-associated protein (D-3-phosphoglycerate dehydrogenase (SERA)), a protein involved in the metabolism and development of the central nervous system [17]; cytoskeleton-associated protein (peripherin (PERI)), a type III intermediate filament (IF) protein that plays a contributory role in motor neuron disease [18, 19]; molecular chaperone (stress-induced-phosphoprotein 1 (STIP1)), a cochaperone intermediating Hsp70/Hsp90 exchange of client proteins and involved in prion protein-mediated neuronal signaling [20] (shown in Table S2); and cytoskeleton-associated protein (TPIS) (shown in Table S3). We found that two proteins, namely, isocitrate dehydrogenase [NADP] cytoplasmic (IDHC), which plays important roles in energy and biosynthesis metabolisms [21], and eukaryotic translation initiation factor 1b (EIF1B), an antiapoptotic protein that protects cells uniquely from Fas-induced apoptosis [22], were commonly changed in various concentrations of DAU-treated N2a/APP cells compared with the vehicle-treated N2a/APP cells (shown in Figure S1B and Table S8). In addition, some common proteins were differentially expressed among the three DAU-treated groups (shown in Figure S1B and Tables S5–6); these included ER stress-associated protein (Far upstream element-binding protein 1 (FUBP1)), a multifunctional DNA- and RNA-binding protein [23]; mitochondrial respiration and metabolism (IDHC) (cytochrome oxidase subunit 5A, mitochondrial (COX5A)), an electron transport chain- (ETC-) related protein (shown in Table S5); ER stress-associated proteins (78 kDa glucose-regulated protein (GRP78), stress-70 protein, and mitochondrial protein (GRP75)); molecular chaperones (heat shock protein HSP 90-beta (HSP90B)), essential molecular chaperone involved in signal transduction, cell cycle control, stress management, and folding, degradation, and transport of proteins [24]; nucleophosmin (NMP), which has a key role activating autophagy induced by nucleolar disruption [25], mitochondrial respiration, and metabolism (IDHC) (shown in Table S6); ER stress-associated protein (protein disulfide-isomerase A3 (PDIA3)), which is mainly involved in the regulation of ER stress (ERS) and protects neurons from ERS-induced apoptosis [26]; and mitochondrial respiration and metabolism (IDHC) (shown in Table S7).

**Figure 6:**
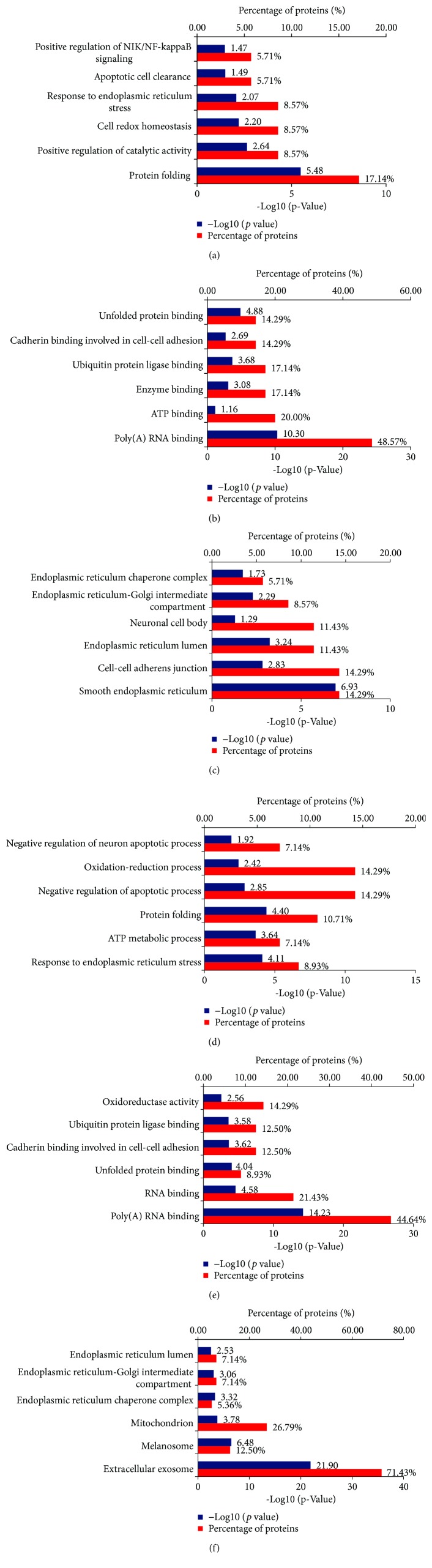
DAVID Gene Ontology enrichment analysis for the dysregulated proteins in N2a/APP cells and DAU-treated N2a/APP cells. (a) Enrichment analysis for the differential proteins by biological process, (b) enrichment analysis for the differential proteins by molecular function, and (c) the cellular component enrichment in Gene Ontology terms of the differentially expressed proteins in N2a/APP cells (when compared with N2a/WT cells). (d) The biological processes, (e) the molecular function, and (f) the cellular component enrichment of the differentially expressed proteins in DAU-treated N2a/APP cells (when compared with untreated N2a/APP cells).

The differentially expressed proteins in DAU-treated N2a/APP cells versus vehicle-treated N2a/APP cells were analyzed and characterized using DAVID. In the vehicle-treated N2a/APP cells, DAVID-defined biological functions for the major clusters of differentially expressed proteins were “protein folding,” “positive regulation of catalytic activity,” and “cell redox homeostasis” (Figure 6(a)), and DAVID-defined protein functions included “poly(A) RNA binding,” “ATP binding,” and “enzyme binding” (Figure 6(b)). DAVID identified DAU-affected cellular compartments as “smooth ER,” “cell-cell adherent junction,” and “ER lumen” (Figure 6(c)). In contrast, DAVID results from the DAU-treated N2a/APP cells were different: the major clusters of differentially expressed proteins were “response to ER stress,” “ATP metabolic process,” and “protein folding,” when categorized according to biological processes of proteins (Figure 6(d)); “poly(A) RNA binding,” “RNA binding,” and “unfolded protein binding,” when categorized according to the molecular function of proteins (Figure 6(e)); and “extracellular exosome,” “melanosome,” and “mitochondria,” when categorized according to cellular component of the proteins (Figure 6(f)).

**Figure 7:**
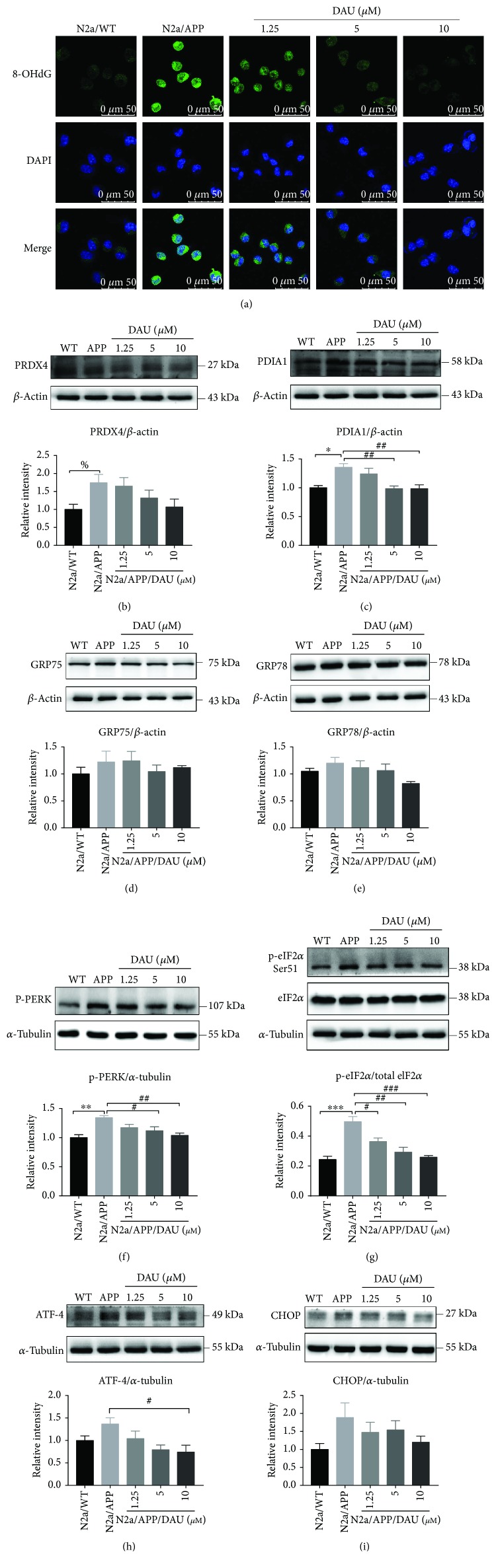
DAU reduced oxidative stress and ER stress of N2a/APP cells. (a) 8-OHdG (in green) immunostaining of N2a/WT and N2a/APP cells. DAPI (in blue) stained the cell nuclei. Proteins PRDX4 (b), PDIA1 (c), GRP75 (d), GRP78 (e), p-PERK (f), p-eIF2*α*, eIF2*α* (g), ATF-4 (h), and CHOP (i) in N2a/WT and N2a/APP cells were determined by Western blot analyses. *β*-Actin was used as a loading control for PRDX4, GRP78, GRP75, and PDIA1. *α*-Tubulin was used as a loading control for ATF-4, CHOP, and p-PERK. Total eIF2*α* was used as a loading control for p-eIF2*α*. *N* = 3. Data show the mean ± SEM. ^%^ *P* = 0.0052, ^∗^ *P* < 0.05, ^∗∗^ *P* < 0.01, and ^∗∗∗^ *P* < 0.001 compared to N2a/WT cells. ^#^ *P* < 0.05, ^##^ *P* < 0.01, and ^###^ *P* < 0.001 compared to untreated N2a/APP.

**Figure 8:**
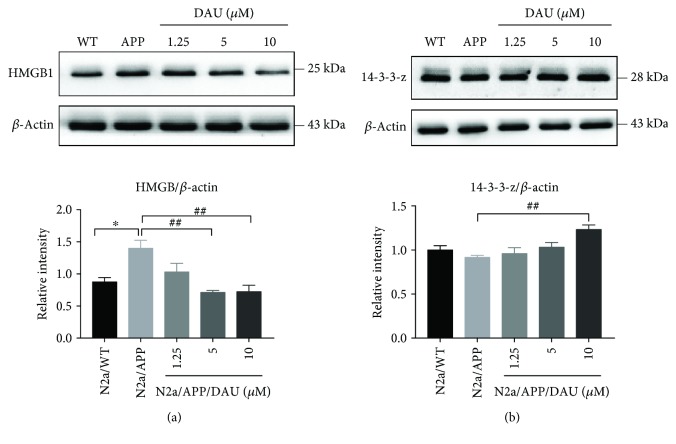
DAU altered the expression of HMGB1 and 14-3-3-z. HMGB1 (a) and 14-3-3-z (b). *N* = 3. Data show the mean ± SEM. ^∗^ *P* < 0.05 compared to N2a/WT cells and ^##^ *P* < 0.01 compared with untreated N2a/APP cells.

To supplement and verify the results of proteomic profiling, we used Western blot analysis to investigate the expression of peroxiredoxin-4 (PRDX4, a member of peroxiredoxin family that regulates redox status of ER) (Figure 7(b), Figure S5A, B, and C); disulfide-isomerase (PDIA1, a member of the PDI family that regulates redox status of ER) (Figure 7(c), Figure S4A, B, and C); GRP75, a mitochondrial chaperone that regulates the mitochondria-associated ER membrane (Figure 7(d), Figure S3A, B, and C); 78 kDa glucose-regulated protein (GRP78, a chaperone binds to ER stress sensor) (Figure 7(e), Figure S2A, B, C, and D); HMGB1 (Figure 8(a), Figure S6A, B, and C), a mediator of neurite degeneration through the identification of the pathological signaling pathway in AD; and 14-3-3 protein zeta/delta (14-3-3-z, a chaperone that involves mitochondrial respiration) (Figure 8(b), Figure S7A, B, and C). The mean level of PRDX4 in N2a/APP cells was significantly higher than that in N2a/WT cells, and the mean level of PRDX4 was reduced in DAU-treated N2a/APP cells. The mean level of PDIA1 in N2a/APP cells was markedly higher than that in N2a/WT cells and reduced in DAU-treated N2a/APP cells. Similar trends were observed for GRP75, GRP78, and HMGB1. The opposite trend was observed for 14-3-3-z.

### 3.5. DAU Reduced Oxidative Stress and ER Stress of N2a/APP Cells

Given the modulation of oxidative stress and ER stress-related proteins by DAU, we investigated the effects of DAU on reactive oxygen species (ROS) and the unfolded protein response (UPR). Shown in Figure 7(a), the staining of 8-oxo-2′-deoxyguanosine (8-OHdG), an indicator of reactive oxygen species (ROS), was significantly higher in N2a/APP cells than in N2a/WT cells. N2a/APP cells treated with DAU at all three concentrations showed significant reductions in 8-OHdG staining compared with that in vehicle-treated N2a/APP cells. We also investigated the protein expressions of most implicated UPR markers, such as GRP75 (Figure 7(d)), GRP78 (Figure 7(e)), phosphorylated pancreatic ER eIF2*α* kinase (p-PERK) (Figure 7(f)), phosphorylated eukaryotic initiation factor-2 alpha (p-eIF2-*α*), eukaryotic translation initiation factor 2 subunits (eIF2*α*) (Figure 7(g)), activating transcription factor-4 (ATF-4) (Figure 7(h)), and transcriptional factor C/EBP homologous protein (CHOP) (Figure 7(i)). The phosphorylation of PERK and eIF2*α* was significantly higher in N2a/APP cells compared with N2a/WT cells, and these phosphorylated proteins were markedly reduced in DAU-treated N2a/APP cells compared with vehicle-treated N2a/APP cells. In addition, the mean levels of GRP75, GRP78, ATF-4, and CHOP were higher in N2a/APP cells compared with N2a/WT cells, and the mean levels of these proteins were reduced in DAU-treated N2a/APP cells compared with vehicle-treated N2a/APP cells.

## 4. Discussion

Dysregulation of neuronal calcium homeostasis plays a crucial role in the progression of AD and therefore is a potential therapeutic target for the treatment of this disease [27]. DAU has been reported to inhibit cellular influx of extracellular Ca^2+^ and restrict the endoplasmic release of Ca^2+^ in various models [28, 29]. Disturbed calcium signaling could lead to ER stress and activate the UPR [30]. Experiments showing DAU's ability to modulate calcium homeostasis have been performed to support the claim that DAU promotes neuronal cell survival [6] and corrects Ca^2+^-mediated arrhythmia in muscle cells [31]. In the present study, DAU suppressed APP processing and A*β* production in a cell model of AD. DAU attenuated tau pathology in N2a/APP cells via the PP2A and p35/25 pathways; this is consistent with a previous report of the effects of DAU on bradykinin-treated N2a cells, which showed that DAU prevents bradykinin-induced alteration of calcium homeostasis and tau hyperphosphorylation in N2a cells [7]. DAU also significantly altered the expression of 85 proteins relevant to oxidative stress, mitochondrial function and metabolism, UPR, and the cytoskeleton.

### 4.1. Oxidative Stress

Oxidative stress-related proteins represent one class of proteins altered by DAU treatment. Oxidative stress is a consequence of calcium influx mediated by *N*-methyl-D-aspartate receptors in AD pathology [32, 33]. Our proteomic profiling showed that some oxidative stress-related proteins were perturbed in N2a/APP cells versus N2a/WT cells. Although protein perturbation does not necessarily link with functional loss of protein, we found that DAU-treated N2a/APP cells retained the expression of peroxiredoxin-2 (PRDX2), PRDX4, and SERA seen in N2a/WT cells (Figure 5). In addition, we observed the level of 8-OHdG was suppressed in the three groups of DAU-treated cells, and the level of 8-OHdG in 5 *μ*M DAU-treated cells was the same as that in N2a/WT cells (shown in Figure 7(a)). This implies that DAU suppressed oxidative stress, which is consistent with previous reports of the antioxidative activities of DAU [34]. The antioxidative effects of DAU could also result from its inhibition of Ca^2+^ influx [7].

### 4.2. Mitochondrial Proteins

Mitochondrial dysfunction is closely linked with oxidative stress in pathological aging and AD [35], and mitochondrial proteins formed another cluster of proteins modified by DAU treatment. In this study, we observed suppression of ATP synthase subunit delta, mitochondrial (ATPD), COX5A, and dihydrolipoyllysine-residue acetyltransferase component of p (ODP2) and enhancement of aldehyde dehydrogenase, mitochondrial (ALDH2), ATP synthase subunit O, mitochondrial (ATPO), electron transfer flavoprotein subunit alpha, mitochondrial (ETFA), IDHC, and transitional endoplasmic reticulum ATPase (TERA) in N2a/APP cells, while protein expression of ACON, ATPD, COX5A, ETFA, IDHC, and ODP2 was retained in DAU-treated cells. This observation may correlate with the report that DAU enhanced the activity of mitochondrial ATPase in a mouse model of cerebral ischemia [36]; however, more functional assays, such as levels of ATP and activities of mitochondrial ATPase, should be included in a future study to validate this correlation. The effect of DAU on mitochondrial function may be related to the modulation of calcium homeostasis by this isoquinoline alkaloid.

### 4.3. Unfolded Protein Response

The unfolded protein response (UPR), an ER stress response to a disturbance in protein folding, is implicated in neurodegenerative diseases [37], including the possibility that accumulation of tau initiates the UPR [37]. UPR-related proteins formed the largest category of proteins modulated by DAU treatment. In the proteomics study, we found that calreticulin (CALR) and Ca^2+^/calmodulin-dependent protein phosphatase had a higher expression in DAU-treated N2a/APP cells versus vehicle-treated N2a/APP cells, suggesting calcium hemostasis was modulated by DAU. In the functional study, we found ER stress markers (Figure 7) were enhanced in N2a/APP cells and suppressed in DAU-treated cells. Calcium homeostasis is also crucial for the function of both ER and mitochondria for the following reasons: (1) Ca^2+^ is predominantly stored in the ER and influx of Ca^2+^ from ER to the mitochondria can be triggered by ER stress [38], and (2) calcium depletion in ER is thought to initiate chronic Ca^2+^ overload in the mitochondria and Bcl-2 dependent apoptosis [39] that DAU suppresses ER stress could be linked to DAU modulation of calcium homeostasis.

Although we categorized the DAU-modulated proteins into oxidative stress, ER stress, and mitochondrial dysfunction, intensive studies reveal these processes are closely related. The oxidative environment in the ER is maintained by the formation of disulfide bonds and the concentration of glutathione, both of which are regulated by the PDI and peroxiredoxin families [40, 41]. Imbalance of redox status or oxidative stress results in ER stress and activates the UPR [30]. During UPR, three classes of ER stress sensors (namely, ATF6, PERK, and IRE1), as well as sensor-bound chaperones (i.e., GRP78), are activated [42–44]. UPR also induces ER stress response genes [44] and proapoptotic transcription factors like CHOP [43]. In the case of AD, the accumulation of A*β* and hyperphosphorylation of tau increase the production of ROS, which results in progressive mitochondrial damage and ER stress via a disturbance of Ca^2+^ homeostasis [45, 46]. Although we did not monitor the Ca^2+^ flow in N2a/APP cells, we investigated the levels of most of the aforementioned proteins, the results of which implied that Ca^2+^ homeostasis may be disturbed in N2a/APP cells. More detailed studies are required to illuminate the mechanisms by which DAU ameliorates mitochondrial dysfunction, ER stress, and oxidative stress by modulating calcium homeostasis. DAU treatment also modified proteins in other categories, including cytoskeleton, molecular chaperone, and signaling proteins. Further investigations on the functions of these proteins might illuminate the novel pharmacological actions of DAU.

### 4.4. Toxicity

The potential toxicity of DAU needs to be addressed. Some toxicological studies of DAU have claimed the isoquinoline alkaloid is biotransformed to a quinone methide intermediate by CYP3A family proteins, and accumulation of the intermediate could then deplete glutathione (GSH) and induce cell apoptosis [47, 48]. Besides, some of the 1,2,3,4-tetrahydroisoquinolines are claimed to be moderate inhibitors of complex I activity and mitochondrial respiration [49]. However, N2a/APP cells could tolerate more than 20 *μ*M DAU without significant loss of cell viability (Figure 1(c)), and N2a/APP cells seem to tolerate higher concentrations of DAU than N2a/WT cells (we observed significant viability loss in N2a/WT cells treated with >10 *μ*M of DAU) (Figure 1(b)). Further investigation into the expression of CYP family proteins in N2a/APP cells is necessary to understand the apparent DAU tolerance of N2a/APP cells. More important, however, is that DAU was protective when N2a/APP cells were treated with concentrations as low as 1.25 *μ*M and, at this concentration, the toxic potential of DAU might be marginal. In a future preclinical study of DAU, dosage should be chosen cautiously to avoid adverse events, including changes in GSH and mitochondrial complex 1 status.

**Figure 9:**
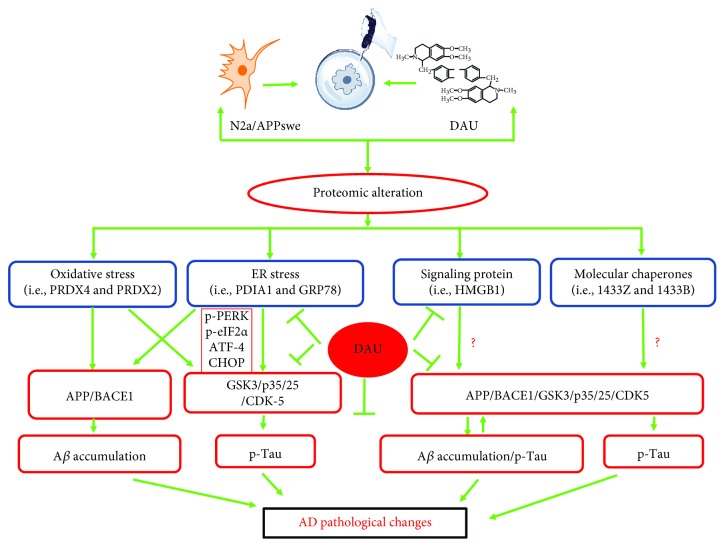
The mode of action of DAU. DAU treatment suppressed AD-related changes, notably A*β* accumulation and tau phosphorylation via APP processing and the CDK5, PP2A, and p35/25 pathways, which may be attributed to the modification of proteins related to functions of oxidative stress, ER stress, molecular chaperones, and signaling protein.

In conclusion, we found DAU treatment attenuated hyperphosphorylation of tau and production of A*β* in N2a/APP cells. DAU also reduced molecular deficits in N2a/APP cells, such as those relating to oxidative stress, ER stress, signaling proteins, and molecular chaperones (summarized in Figure 9). Although N2a/APP cells tolerated higher concentrations of DAU, we recommend focus on the lowest effective concentrations and doses of DAU to avoid adverse events in cell culture and animal studies, respectively.

## Figures and Tables

**Table 1:** The primary antibodies used in this study.

Antibody	Cat.	RRID	Type	Dilution	Source
t-APP	ab32136	AB_2289606	Rabbit	1 : 3000	Abcam
p-APP	#6986	BDSC_6986	Rabbit	1 : 1000	Cell Signaling
sAPP*α*	11088	Unknown	Mouse	1 : 50	Immuno-Biological
sAPP*β*	10321	Unknown	Mouse	1 : 50	Immuno-Biological
BACE1	#5606	AB_1903900	Rabbit	1 : 1000	Cell Signaling
PS1	#5643	AB_10706356	Rabbit	1 : 1000	Cell Signaling
pS396	ab109390	AB_10860822	Rabbit	1 : 20000	Abcam
pS404	sc-12952	RRID:AB_656753	Goat	1 : 3000	Santa Cruz
pT231	355200	AB_2533210	Mouse	1 : 1000	Thermo Fisher
pS262	44750G	AB_2533743	Rabbit	1 : 1000	Thermo Fisher
Tau 1	MAB3420	AB_94855	Mouse	1 : 200000	Millipore
Tau 5	ab80579	AB_1603723	Mouse	1 : 3000	Abcam
p-GSK3*α*/*β*	#9331	AB_329830	Rabbit	1 : 1000	Cell Signaling
GSK3*α*/*β*	#5676	AB_10547140	Rabbit	1 : 1000	Cell Signaling
p-PP2A (Y307)	AF3989	AB_2169636	Rabbit	1 : 1000	R&D Systems
PP2A	#2259	AB_10695752	Rabbit	1 : 1000	Cell Signaling
p35/25	#2680	AB_1078214	Rabbit	1 : 1000	Cell Signaling
CDK5	ab40773	AB_726779	Rabbit	1 : 3000	Abcam
GRP78	sc-376768	Unknown	Mouse	1 : 1000	Santa Cruz
GRP75	sc-133137	AB_2120468	Mouse	1 : 1000	Santa Cruz
PDIA1	ab2792	AB_303304	Mouse	1 : 1000	Abcam
HMGB1	ab79823	AB_1603373	Rabbit	1 : 50000	Abcam
14-3-3-z	ab155037	Unknown	Rabbit	1 : 3000	Abcam
PRDX4	ab16943	AB_443567	Rabbit	1 : 1000	Abcam
8-OHdG	ab10802	AB_297482	Goat	1 : 400	Abcam
p-PERK	#3179	AB_2095853	Rabbit	1 : 1000	Cell Signaling
p-eIF2*α*	#3597	RRID:AB_390740	Rabbit	1 : 1000	Cell Signaling
eIF2*α*	#5324	AB_10692650	Rabbit	1 : 1000	Cell Signaling
ATF-4	#11815	AB_2616025	Mouse	1 : 1000	Cell Signaling
CHOP	#2895	AB_2089254	Rabbit	1 : 1000	Cell Signaling
*β*-Actin	sc-47778	AB_626632	Mouse	1 : 3000	Santa Cruz
*α*-Tubulin	sc-73242	AB_1130901	Mouse	1 : 3000	Santa Cruz

## Abbreviations

AD: Alzheimer's disease
DAU: Dauricine
2D-DIGE: Two-dimensional fluorescence difference gel electrophoresis
APP: Amyloid precursor protein
sAPP*β*: *β*-Secretase-cleaved amyloid precursor protein
sAPP*α*: *α*-Secretase-cleaved amyloid precursor protein
BACE1: *β*-Site APP cleaving enzyme 1
GSK-3*α*: Glycogen synthase kinase-3*α*
GSK-3*β*: Glycogen synthase kinase-3*β*
CDK5: Cyclin-dependent kinase 5
N2a/WT: Wild-type murine neuroblastoma
N2a/APP: Murine neuroblastoma N2a cells stably transfected with Swedish mutant of amyloid precursor protein
PP2A: Protein phosphatase2A
HEK293/Tau: Human embryonic kidney 293 cells stably transfected with tau protein
ER stress: Endoplasmic reticulum stress
A*β*_1–42_: Amyloid-*β* 1–42
A*β*_1–40_: Amyloid-*β* 1–40
GO: Gene ontology
BP: Biological processes
MF: Molecular functions
CC: Cellular components
PS1: Presenilin 1
HMGB1: High-mobility group protein B1
HSP7C: Heat shock cognate 71 kDa protein
TCTP: Translationally controlled tumor protein
PDIA6: Protein disulfide-isomerase A6
SERA: D-3-Phosphoglycerate dehydrogenase
PERI: Peripherin
STIP1: Stress-induced phosphoprotein 1
IDHC: Isocitrate dehydrogenase [NADP] cytoplasmic
EIF1B: Eukaryotic translation initiation factor 1b
FUBP1: Far upstream element-binding protein 1
COX5A: Cytochrome c oxidase subunit 5A, mitochondrial
GRP78: 78 kDa glucose-regulated protein
GRP75: Stress-70 protein, mitochondrial protein
HSP90B: Heat shock protein HSP90-beta
NMP: Nucleophosmin
PDIA3: Protein disulfide-isomerase A3
PRDX4: Peroxiredoxin-4
PDIA1: Disulfide-isomerase
14-3-3-z: 14-3-3 protein zeta/delta
ROS: Reactive oxygen species
UPR: Unfolded protein response
8-OHdG: 8-Oxo-2′-deoxyguanosine
ATF-4: Activating transcription factor-4
CHOP: Transcriptional factor C/EBP homologous protein
p-eIF2*α*: Phosphorylated eukaryotic initiation factor-2 alpha
p-PERK: Pancreatic ER eIF2*α* kinase
UPR: Unfolded protein response
PRDX2: Peroxiredoxin-2
ATPD: ATP synthase subunit delta, mitochondrial
ODP2: Dihydrolipoyllysine-residue acetyltransferase component of p
ALDH2: Aldehyde dehydrogenase, mitochondrial
ATPO: ATP synthase subunit O, mitochondrial
ETFA: Electron transfer flavoprotein subunit alpha, mitochondrial
TERA: Transitional endoplasmic reticulum ATPase.

## Supplementary Materials

Supplementary Materials Figure S1: common proteins differentially expressed among the different comparison groups. Figure S2–S7: MALDI-TOF-MS map of GRP78 (Figure S2), GRP75 (Figure S3), PDIA1 (Figure S4), PRDX4 (Figure S5), HMGB1 (Figure S6), and 14-3-3-z (Figure S7). Table S1–S8: list of common proteins differentially expressed among the different comparison groups.
