# Supplementary material for: The Isoquinoline Alkaloid Dauricine Targets Multiple Molecular Pathways to Ameliorate Alzheimer-Like Pathological Changes In Vitro
Source: Oxid Med Cell Longev. 2018 Jul 2;2018:2025914. doi: 10.1155/2018/2025914 (PMC6051032; doi:10.1155/2018/2025914)
Supplement: Supplementary Materials — Figure S1: common proteins differentially expressed among the different comparison groups. Figure S2–S7: MALDI-TOF-MS map of GRP78 (Figure S2), GRP75 (Figure S3), PDIA1 (Figure S4), PRDX4 (Figure S5), HMGB1 (Figure S6), and 14-3-3-z (Figure S7). Table S1–S8: list of common proteins differentially expressed among the different comparison groups. [file 2025914.f1.docx]

**Supporting information**

**Figure S1**


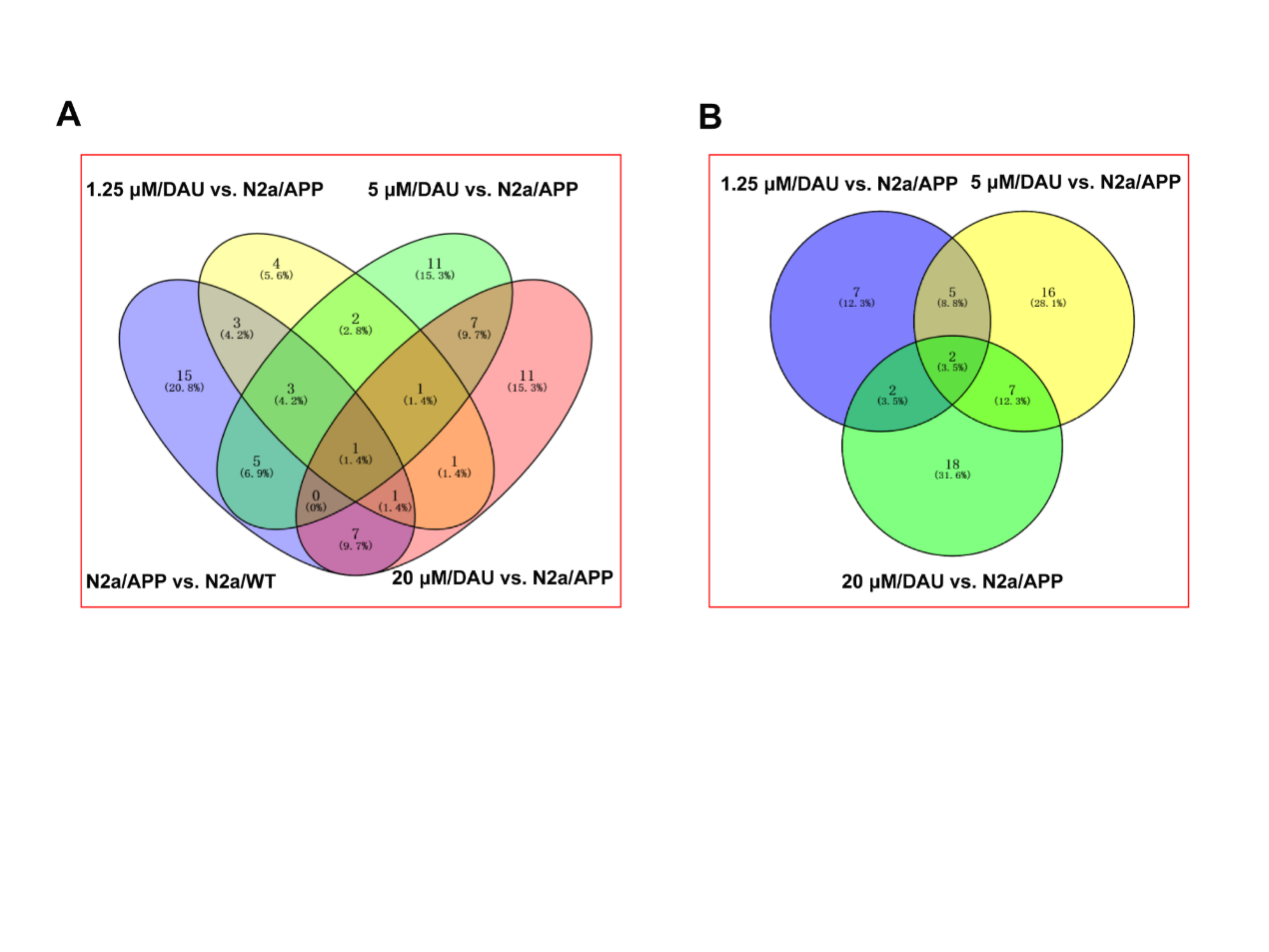


One protein (EIF1B) (A) was changed in four comparison groups. Two proteins (IDHC, EIF1B) (B) were changed in various concertrations of DAU-treated N2a/APP cells compared with untreated N2a/APP cells.

**Figure S2**


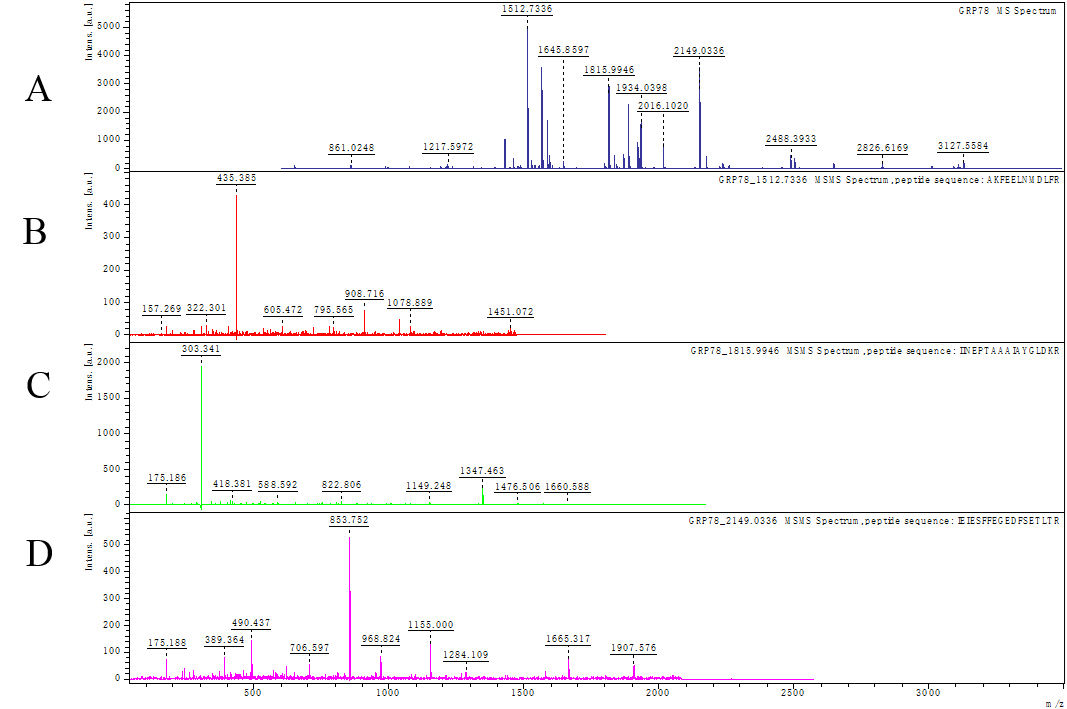


The MALDI-TOF-MS map of GRP78 (A); MS/MS spectrum of fingerprints peptide of GRP78 (B) m/z 1512.7336, (C)m/z 1815.9946, (D) m/z 2149.0336.

**Figure S3**


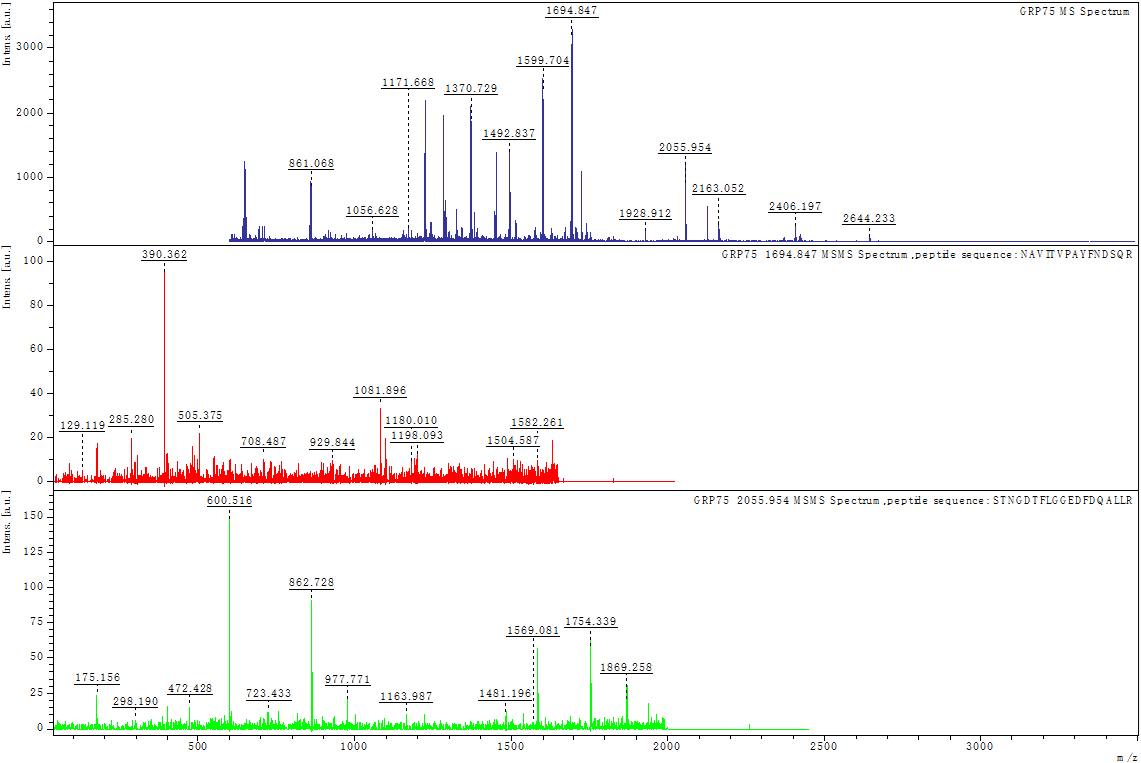


C

B

A

The MALDI-TOF-MS map of GRP75 (A); MS/MS spectrum of fingerprints peptide of GRP75 (B) m/z 1694.847, (C) m/z 2055.954.

**Figure S4**


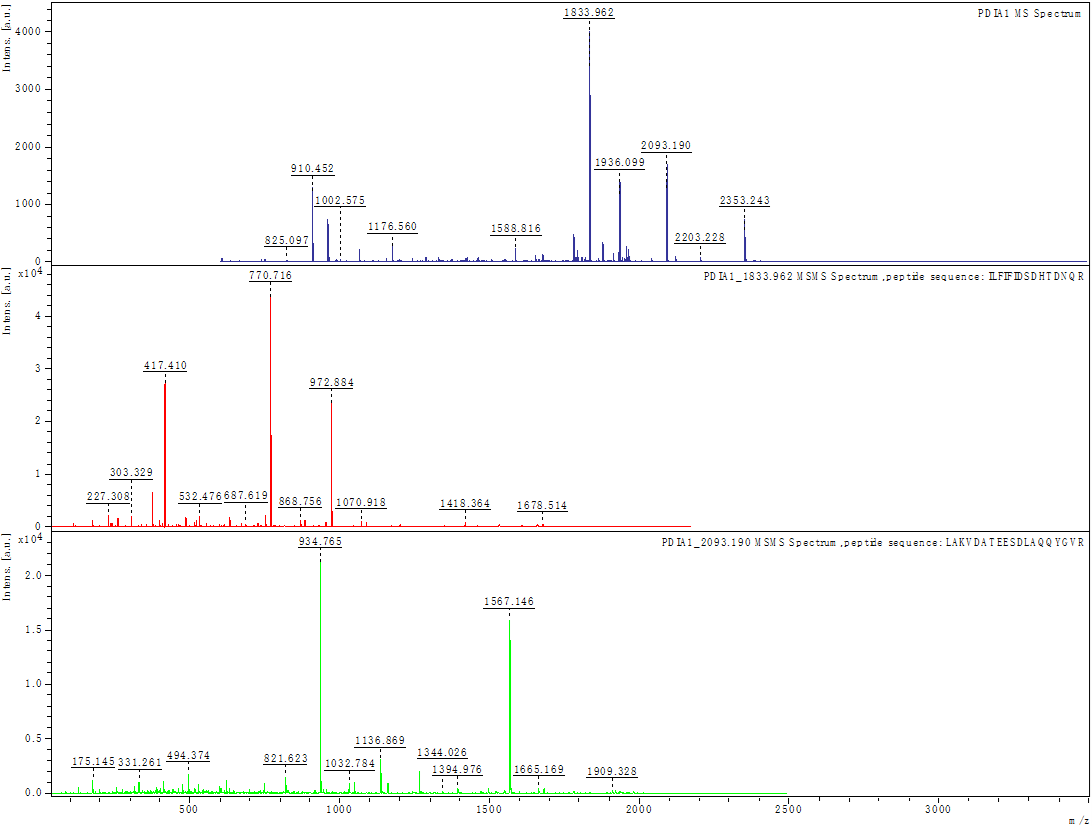


A

C

B

The MALDI-TOF-MS map of PDIA1 (A); MS/MS spectrum of fingerprints peptide of PDIA1 (B) m/z 1833.962, (C) m/z 2093.190.

**Figure S5**


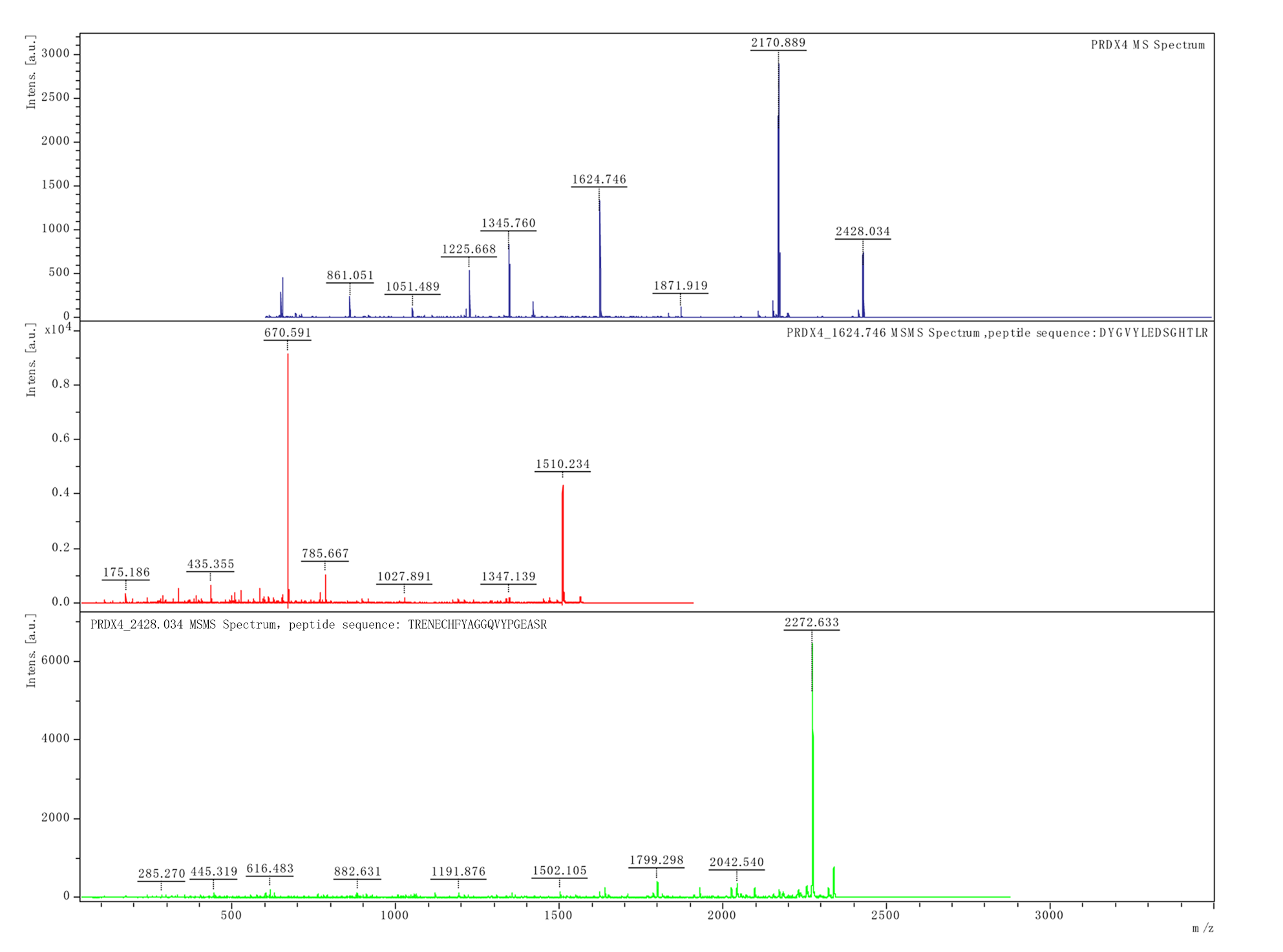


C

B

A

The MALDI-TOF-MS map of PRDX4 (A); MS/MS spectrum of fingerprints peptide of PRDX4 (B) m/z 1624.746, (C) m/z 2428.034.

**Figure S6**


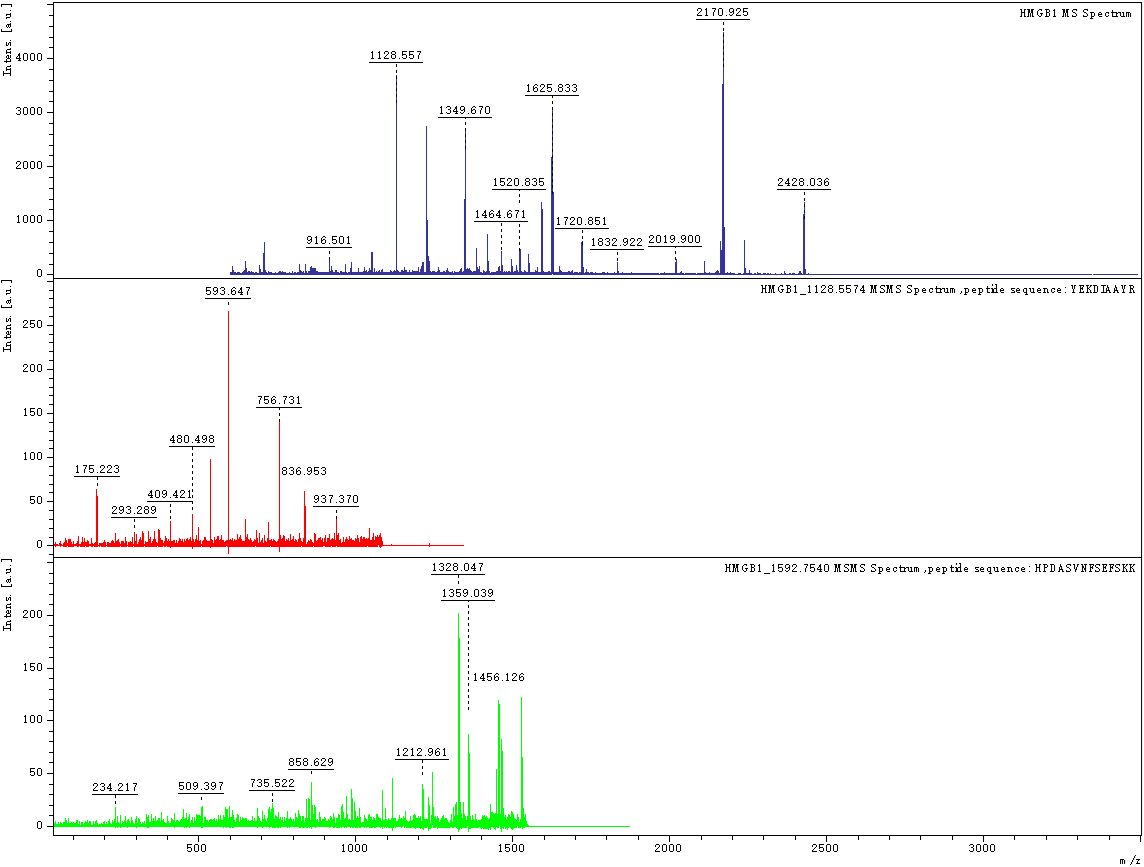


C

B

A

The MALDI-TOF-MS map of HMGB1 (A); MS/MS spectrum of fingerprints peptide of HMGB1 (B) m/z 1128.557, (C) m/z 1592.7540.

**Figure S7**


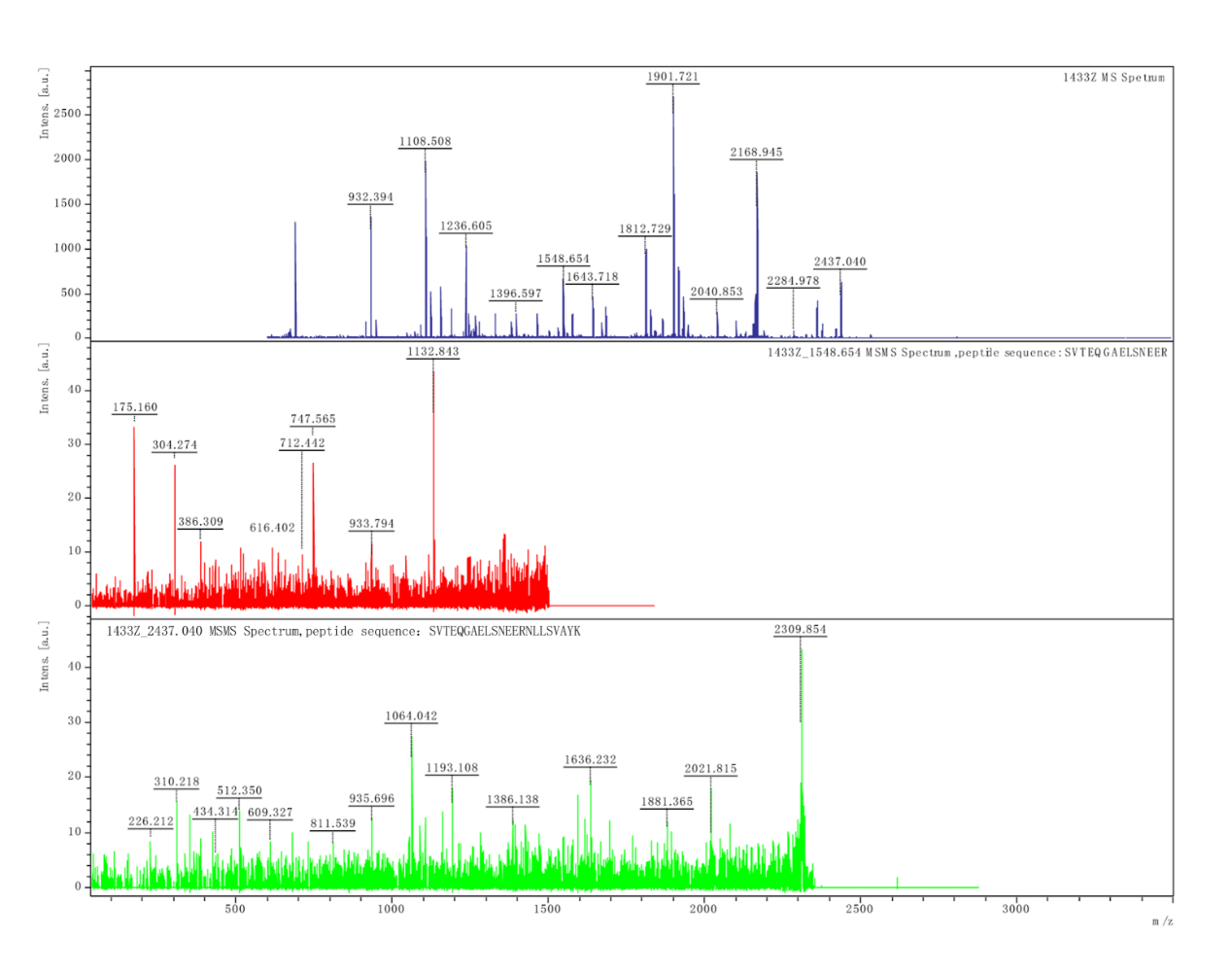


C

B

A

The MALDI-TOF-MS map of 1433Z (A); MS/MS spectrum of fingerprints peptide of 1433Z (B) m/z 1548.654, (C) m/z 2437.040.

| Uniport | Protein name | Ratio | |
| --- | --- | --- | --- |
|  |  | N2a/APP vs. N2a/WT & 1.25 µM/DAU vs. N2a/APP | |
| **Signaling protein** | |  |  |
| P63158 | High mobility group protein B1 | 5.43 | -1.33 |
| **Molecular chaperone** | |  |  |
| P63017 | Heat shock cognate 71 kDa protein | 1.56 | 1.24 |
| **Cytoskeleton-associated protein** | |  |  |
| P63028 | Translationally-controlled tumor protein | 1.65 | 1.12 |

**TableS1 Liu et al., 2017**

| Uniport | Protein name | Ratio | |
| --- | --- | --- | --- |
|  |  | N2a/APP vs. N2a/WT & 5 µM/DAU vs. N2a/APP | |
| **ER stress-associated protein** | |  |  |
| Q922R8 | Protein disulfide-isomerase A6 | 1.39 | 1.05 |
| **Oxidative stress-associated protein** | |  |  |
| Q61753 | D-3-phosphoglycerate dehydrogenase | -1.18 | 1.27 |
| **Cytoskeleton-associated protein** | |  |  |
| P15331 | Peripherin | 1.24 | 1.07 |
| **Molecular chaperone** | |  |  |
| Q60864 | Stress-induced-phosphoprotein 1 | -1.15 | 1.22 |
| **Other** |  |  |  |
| Q61206 | Platelet-activating factor acetylhydrolase IB subunit beta | -1.21 | 2.2 |

**TableS2 Liu et al., 2017**

| Uniport | Protein name | Ratio | |
| --- | --- | --- | --- |
|  |  | N2a/APP vs. N2a/WT & 20 µM/DAU vs. N2a/APP | |
| **Cytoskeleton-associated protein** | |  |  |
| P17751 | Triosephosphate isomerase | 1.26 | -1.38 |
| **Others** |  |  |  |
| Q3THK7 | GMP synthase [glutamine-hydrolyzing] | 2.52 | -1.44 |
| Q8R081 | Heterogeneous nuclear ribonucleoprotein L | -1.5 | -1.5 |
| P63242 | Eukaryotic translation initiation factor 5A-1 | -1.6 | -1.14 |
| P60335 | Poly(rC)-binding protein 1 | -1.19 | -1.29 |
| P54227 | Stathmin | -1.38 | -1.18 |

**TableS3 Liu et al., 2017**

| Uniport | Protein name | Ratio | | | |
| --- | --- | --- | --- | --- | --- |
|  |  | N2a/APP vs. N2a/WT & 1.25µM/DAU vs. N2a/APP & 5 µM/DAU vs. N2a/APP & 20 µM/DAU vs. N2a/APP | | | |
| **Other** |  |  |  |  |  |
| Q9CXU9 | Eukaryotic translation initiation factor 1b | 1.2 | 1.13 | -1.12 | 1.18 |

**TableS4 Liu et al., 2017**

| Uniport | Protein name | Ratio | |
| --- | --- | --- | --- |
|  |  | 20 µM/DAU vs. N2a/APP & 5 µM/DAU vs. N2a/APP | |
| **ER stress-associated protein** | |  |  |
| Q91WJ8 | Far upstream element-binding protein 1 | -1.25 | 1.11 |
| **Mitochondrial respiration and metabolism** | |  |  |
| O88844 | Isocitrate dehydrogenase [NADP] cytoplasmic | -1.48 | -1.18 |
| P12787 | Cytochrome c oxidase subunit 5A, mitochondrial | 1.29 | 1.29 |
| **Others** |  |  |  |
| Q99LC5 | Electron transfer flavoprotein subunit alpha, mitochondrial | -1.56 | -1.19 |
| Q9WUK2 | Eukaryotic translation initiation factor 4H | -1.33 | -1.16 |
| P70696 | Histone H2B type 1-A | 1.40 | 1.14 |
| P24547 | Inosine-5'-monophosphate dehydrogenase 2 | 1.41 | -1.16 |
| P14869 | 60S acidic ribosomal protein P0 | -1.45 | 1.32 |
| Q9CXU9 | Eukaryotic translation initiation factor 1b | 1.20 | -1.12 |

**TableS5 Liu et al., 2017**

| Uniport | Protein name | Ratio | |
| --- | --- | --- | --- |
|  |  | 5 µM/DAU vs. N2a/APP & 1.25 µM/DAU vs. N2a/APP | |
| **ER stress-associated proteins** | |  |  |
| P38647 | Stress-70 protein, mitochondrial | 1.09 | 1.09 |
| P20029 | 78 kDa glucose-regulated protein | 1.07 | 1.12 |
| **Molecular chaperones** | |  |  |
| P11499 | Heat shock protein HSP 90-beta | 1.13 | 1.22 |
| Q61937 | Nucleophosmin | -1.3 | -1.06 |
| **Mitochondrial respiration and metabolism** | |  |  |
| O88844 | Isocitrate dehydrogenase [NADP] cytoplasmic | -1.18 | -1.09 |
| **Others** |  |  |  |
| P47753 | F-actin-capping protein subunit alpha-1 | -1.48 | -1.54 |
| Q9CXU9 | Eukaryotic translation initiation factor 1b | -1.12 | 1.13 |

**TableS6 Liu et al., 2017**

| Uniport | Protein name | Ratio | |
| --- | --- | --- | --- |
|  |  | 20 µM/DAU vs. N2a/APP & 1.25 µM/DAU vs. N2a/APP | |
| **ER stress-associated protein** | |  |  |
| P27773 | Protein disulfide-isomerase A3 | -1.16 | 1.05 |
| **Mitochondrial respiration and metabolism** | |  |  |
| O88844 | Isocitrate dehydrogenase [NADP] cytoplasmic | -1.48 | -1.09 |
| **Others** |  |  |  |
| P16045 | Galectin-1 | 5.25 | -1.27 |
| Q9CXU9 | Eukaryotic translation initiation factor 1b | 1.2 | 1.13 |

**TableS7 Liu et al., 2017**

| Uniport | Protein name | Ratio | | |
| --- | --- | --- | --- | --- |
|  |  | 20 µM/DAU vs. N2a/APP & 5 µM/DAU vs. N2a/APP & 1.25 µM/DAU vs. N2a/APP | | |
| **Mitochondrial respiration and metabolism** | |  |  |  |
| O88844 | Isocitrate dehydrogenase [NADP] cytoplasmic | -1.48 | -1.18 | -1.09 |
| **Other** |  |  |  |  |
| Q9CXU9 | Eukaryotic translation initiation factor 1b | 1.2 | -1.12 | 1.13 |

**TableS8 Liu et al., 2017**
